# Engineering nanopores in hard carbon for high-energy sodium-ion batteries

**DOI:** 10.1093/nsr/nwag187

**Published:** 2026-03-25

**Authors:** Chuanlian Xiao, Chuanhai Gan, Joachim Maier

**Affiliations:** Max Planck Institute for Solid State Research, Germany; Max Planck Institute for Solid State Research, Germany; Max Planck Institute for Solid State Research, Germany

The growing demand for large-scale energy storage and the comparatively limited abundance of lithium has stimulated increased interest in sodium-ion batteries (SIBs), which are considered to be promising alternatives to lithium-ion systems due to the ubiquity and low cost of sodium resources [1]. However, the development of high-energy SIBs remains strongly dependent on improving choice and performance of the anode material. Among available candidates, hard carbon is currently the most practical anode [2], yet its sodium storage mechanism remains complex and not fully understood. In particular, the origin of the low-voltage plateau capacity—commonly attributed to the filling of nanopores—poses challenges for achieving high reversible capacity, high initial Coulombic efficiency and long-term cycling stability.

**Figure 1.**
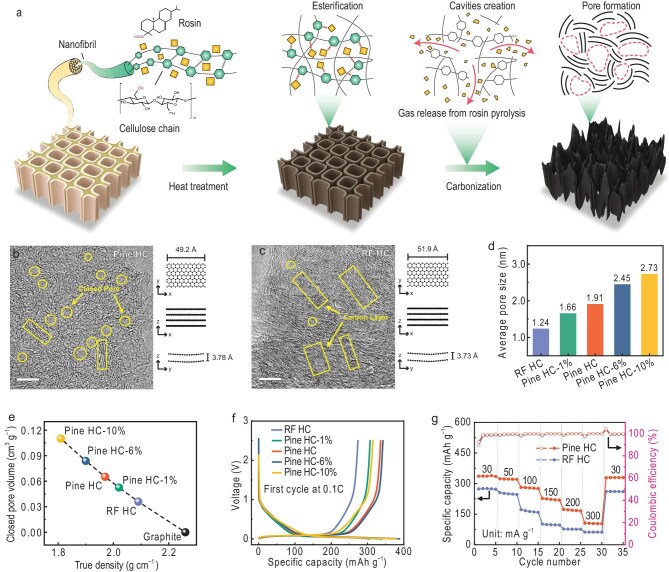
(a) Schematic illustration of the role of rosin in promoting micropore formation in biomass-derived hard carbon (HC). For clarity, only the cellulose chain is shown; however, hydroxyl groups in hemicellulose and lignin can also undergo esterification with rosin. (b, c) High-resolution transmission electron microscopy (HRTEM) images of Pine HC (b) and rosin-free (RF) HC (c). Scale bars: 5 nm. (d) Average closed-pore size derived from small-angle X-ray scattering (SAXS) profiles. (e) True (skeletal) density and the corresponding closed-pore volume of Pine HCs, RF HC and graphite. (f) Galvanostatic discharge–charge profiles of Pine HCs and RF HC at 30 mA g^−1^. (g) Rate performance of Pine HC and RF HC at current densities ranging from 30 to 300 mA g^−1^. Reproduced from ref. [3] with permission.

In a recent study, Yu and co-workers report an exciting strategy to engineer the nanoporous structure of hard carbon to enhance sodium storage performance and enable practical high-energy SIBs [3]. By introducing rosin acids via esterification into the biomass followed by pyrolysis (Fig. 1a), the authors achieve quantitative control over closed nanopores within the carbon matrix (Fig. 1b–e). These nanopores act as effective sodium storage sites and significantly increase the plateau capacity of the material. As a result, the optimized hard carbon exhibits improved reversible capacity (Fig. 1f and g) and enables sodium-ion pouch cells with remarkable overall energy densities exceeding 200 Wh kg^−1^ (if pine wood is used as precursor). What is more, the strategy to use plants as carbon source is of special interest from an ecological point of view.

The preparation strategy is conceptually simple but effective. To reiterate, rosin acid interacts with selected biomasses (typically pine wood but also litchi, camphor, etc.) whereby the subsequent carbonization process yields a tailored distribution of closed nanopores in the final ‘hard carbon’ (Fig. 1a). Structural characterization—including X-ray scattering, spectroscopy and electron microscopy—confirms that the pore architecture plays a central role in regulating sodium storage behavior (Fig. 1b–g).

Beyond the materials engineering aspect, the work raises intriguing mechanistic questions. In particular, how sodium ions access and occupy closed nanopores remains an open question. Understanding the transport pathway into these confined spaces could provide deeper insight into the plateau storage mechanism in hard carbon. Such processes may even involve cooperative ion-electron transport in a ‘job-sharing’ way [4]. It seems that the limited capacity retention remains a key point to be addressed in future research. Does the reason lie in the structural variation as far as the pore pattern is concerned, or is it mainly due to interfacial problems? Answering these questions could lead to a straightforward improvement of the performance.

In short, this important work highlights the significance of nanoscale pore engineering in controlling sodium storage mechanisms in hard carbon anodes. The strategy offers a practical pathway toward high-energy SIBs and may inspire future studies aimed at elucidating ion transport and storage in nanoporous carbon frameworks.
